#  Editorial: Data gathering, quality and security in intelligent phytoprotection

**DOI:** 10.3389/fpls.2022.1010525

**Published:** 2022-09-05

**Authors:** Jiachen Yang, Qinggang Meng, Wen Lu, Longsheng Fu, Baohua Zhang

**Affiliations:** ^1^School of Electrical and Information Engineering, Tianjin University, Tainjin, China; ^2^School of Computer Science and Informatics, Loughborough University, Loughborough, United Kingdom; ^3^School of Electronic Engineering, Xidian University, Xi'an, China; ^4^College of Mechanical and Electronic Engineering, Northwest A&F University, Xianyang, China; ^5^College of Artificial Intelligence, Nanjing Agricultural University, Nanjing, China

**Keywords:** plant disease recognition, pest detection, machine learning, agricultural machinery, data mining

## 1. Background

Intelligent phytoprotection systems use modern information and communication technologies to protect crop growth while optimizing the required human labor. A fundamental challenge is to understand the complex biological environment through the set of sensors and develop intelligent phytoprotection applications at the system level. Recent technological advances in machine vision, data mining, image processing, cloud computing, edge computing, and Internet of Things (IoT) have significantly enabled AI-driven smart crop protection systems. Most of the current studies rely on large labeled datasets with high cost of data acquisition and annotation. As a necessary addition in the community, few-shot learning aims to learn from limited labeled data to obtain generalized models. Therefore, Internet of Things (IoT) data collection and processing is crucial, while data quality and security are essential for Deep Learning based on Big Data and Little-shot learning based on limited data in AI-driven intelligent crop protection. Instead of using grossly large amounts of redundant data, data information analysis and efficient learning methods based on limited data may be more useful for certain real-world tasks in intelligent crop protection. The future smart agricultural systems should use limited high-quality data and seriously consider data security, which will benefit the use of smart crop protection and agricultural equipment.

This Research Topic aims to collect researches focusing on data gathering, quality, and security in AI-driven intelligent phytoprotection systems, especially advanced few-shot learning and deep learning based on data mining and information evaluation. The scope of this topic includes: (i) Data gathering from smart IoT sensors in intelligent phytoprotection systems. (ii) Data mining and information analysis for AI-driven phytoprotection systems. (iii) Fusion and processing of multi-source data for deep learning in phytoprotection systems. (iv) Specific applications, e.g., plant pest and disease detection, yield prediction, irrigation strategy, online agricultural data acquisition, smart agricultural machinery, data communication, etc.

## 2. Summary

Li and Chao introduced an embedding range judgment method in the feature space. The selected high-quality data with less quantity can reach the same performance with all training data in some computer vision tasks. In addition, they also found that limited high-quality data can be better than many bad data in the final test effect, and the comparison of relevant experiments is very significant.

Li and Chao reported an index to distinguish good data from bad data from the perspective of information, which is named effective distance entropy. The results of relevant comparative experiments and ablation experiments showed that the distance entropy method proposed by them is effective and robust in various applications. This work focused on the image quality problem centered on the amount of information, which had a certain enlightenment for the follow-up information mining.

D'Ascenzo et al. reported a new digital signal processing method, which uses sparse data assimilation technology to directly calculate the dynamic evolution of noise correlation between estimated variables and measured variables. The sequential time stepping program estimated the spatial distribution based on TAC signals.

Yu et al. proposed a semantic segmentation method of U-Net with improved structure and SE module based on channel attention mechanism. This method first extracted broad-leaved weeds through image morphological operations, and generated segmentation images of soybean plants, weeds, and broad-leaved weeds. Based on the above pretreatment classification, the identified weeds were further refined into various types of weeds, which provides strong support for intelligent, accurate, and controllable weed treatment.

Nie et al. put forward a prediction model of liquid magnetization series data, which was composed of condition generative adversarial network and projection gradient descent algorithm. A large number of experiments had been carried out in this work. The relevant experimental results showed that when the data samples were limited, the combination of random gradient algorithm and conditional generative adversarial network could obtain a generation closer to the expected effect.

Lv et al. improved the yolov3 model, and combines this method with image enhancement to achieve more accurate detection of crop pests in the real agricultural environment. Experiment results showed that the mAP and mRecall of the improved yolov3 model were improved by 6.3 and 4.61%. Relevant experiments showed that the detection performance of this method was better than other methods, which showed that this method had achieved the expected effect, and provided a reliable model for the intelligent monitoring of corn pests.

## Author contributions

All authors contributed to the Research Topic and wrote and edited the editorial. All authors contributed to the article and approved the submitted version.

## Conflict of interest

The authors declare that the research was conducted in the absence of any commercial or financial relationships that could be construed as a potential conflict of interest.

## Publisher's note

All claims expressed in this article are solely those of the authors and do not necessarily represent those of their affiliated organizations, or those of the publisher, the editors and the reviewers. Any product that may be evaluated in this article, or claim that may be made by its manufacturer, is not guaranteed or endorsed by the publisher.

